# The CT pulmonary vascular parameters and disease severity in COPD patients on acute exacerbation: a correlation analysis

**DOI:** 10.1186/s12890-020-01374-6

**Published:** 2021-01-20

**Authors:** Tao Yang, Chihua Chen, Zhongyuanlong Chen

**Affiliations:** 1 Imaging Department, Linyi Central Hospital, Linyi, 276400 Shandong China; 2 Radiology Department, Hubei Provincial Hospital of Integrated Chinese & Western Medicine, Wuhan, 430015 Hubei China; 3 Department of Radiology, Chest Hospital of Xinjiang Uygur Autonomous Region of the PRC, No. 106, Yan’an road, Urumqi, 830049 Xinjiang China

**Keywords:** CT, Parameter, Vascular, COPD, Diagnosis

## Abstract

**Background:**

It is necessary to analyze the CT pulmonary vascular parameters and disease severity in chronic obstructive pulmonary disease (COPD) patients to provide evidence support for the management of COPD.

**Methods:**

COPD patients on acute exacerbation admitted to our hospital from COPD patients from January 2019 to March 2020 was selected. The characteristics and ratio of the cross-sectional area (CSA) of small pulmonary vessels to the total area of the lung field, and the ratio of pulmonary artery and aorta (PA/A) cross-sectional diameter in patients with COPD were analyzed.

**Results:**

A total of 128 COPD patients were included. There were significant differences in the duration of COPD, smoking history, the PaO_2_, PaCO_2_, pH, and FEV1, FVC and FEV1/FVC among COPD patients with different severity (all p < 0.05). The duration of COPD, smoking, PaO_2_, PaCO_2_, CSA and PA/A were correlated with the COPD severity (all *p* < 0.05). Both CSA, PA/A were correlated with post BD FEV1 (all *p* < 0.05). The cutoff value of CSA and PA/A for the diagnosis of severe COPD was 0.61 and 0.87 respectively, and the AUC of CSA and PA/A for the diagnosis of severe COPD was 0.724 and 0.782 respectively.

**Conclusions:**

Patients with CSA ≤ 0.61 and PA/A ≥ 0.87 may have higher risks for severe COPD, and more studies are needed in the future to further elucidate the management of COPD.

## Background

Chronic obstructive pulmonary disease (COPD) is a common chronic inflammatory lung disease in the elder patient, which is characterized by persistent respiratory symptoms and restricted airflow. Respiratory symptoms and airflow obstruction are caused by toxic particles or gases, which cause abnormalities in the airways and/or alveoli [1, 2]. Cigarette smoke has been reported to be the most common risk factor for COPD [3]. At present, the clinical diagnosis and classification of COPD is mainly based on lung function tests [4]. Pulmonary function test is a non-invasive test method, which is the main basis for diagnosis and evaluation of COPD. After inhaling bronchodilator FEV1/FVC (forced expiratory volume in 1 s/forced vital capacity) < 0.70, indicating continuous airflow obstruction, which is the diagnostic criterion for COPD [5].

However, the pulmonary function test may also have some limitations: firstly, some patients may have bias in the test results due to different educational levels and understanding [6]. Besides, some patients have aggravated illness or there are relative and absolute contraindications [7]. For example, patients with acute exacerbation of pneumothorax, myocardial infarction, etc., cannot tolerate the examination [8]. Furthermore, at early stage of lung tissue and small airway destruction, the lung function test is not easy to detect the existence of COPD. The test may only reflect the state of lung function, but the location of the lesion needs to be further detected in the pulmonary detection [9]. Therefore, it is necessary to seek new means to early detect and reflect the progress of COPD's disease changes.

Pulmonary vascular change is an important pathophysiological characteristic of COPD. On the one hand, COPD causes the destruction of the lung parenchyma, which leads to the loss of the attachment of the alveoli and the small airways and the reduction of the elasticity of the lungs [10]. Previous studies [11–13] have shown that pulmonary vascular parameters of high-resolution computed tomography (HRCT) of the chest, such as the ratio of the cross-sectional area of small pulmonary vessels to the total area of the lung field (%cross-sectional area, CSA), the ratio of pulmonary artery and aorta(PA/A) cross-sectional diameter are potentially related to the pulmonary function indexes. Therefore, we attempted to conduct this retrospective study to analyze the potential relationship of HRCT pulmonary vascular parameters and disease severity in COPD patients, to provide evidence for the treatment of COPD.

## Methods

Our study was a retrospective analysis. This present study had been verified and approved by the ethics committee of Chest Hospital of Xinjiang Uygur Autonomous region of the PRC (approval number: 20190112-3a), and written informed consents had been obtained from all the included patients.

### Patients

COPD patients from January 2019 to March 2020 on acute exacerbation treated in the Department of Respiratory Medicine of our hospital were identified as potential participants. The inclusion criteria were: (1) adult patients with age ≥ 40 years old and patients had smoking history ≥ 10 pack years; (2) The COPD diagnosis met the diagnostic criteria of the Global Initiative for Chronic Obstructive Lung Disease (GOLD) guidelines (2020 edition) [14]; (3) All patients underwent HRCT examination upon admission; (4) the patient has been informed and agreed to participate in this study. The exclusion criteria were: (1) The patient complicated with other lung diseases such as pneumonia, lung cancer, pulmonary embolism or pulmonary hypertension et al. that might affect pulmonary blood flows; (2) patient with history of lung surgery; (3) patients refused to participate in this study.

Based on the Chinese Expert Consensus on the Diagnosis and Treatment of COPD [15], and according to its clinical manifestations and arterial blood gas analysis, the patients were divided into mild (no respiratory failure, the forced expiratory volume in the first second is less than 70% of the vital capacity, but FEV1 was greater than or equal to 80% of the predicted value), moderate (acute respiratory failure but no life-threatening, FEV1 was greater than or equal to 50% and less than 80% of the expected value) and severe (acute respiratory failure and life-threatening, FEV1 was greater than or equal to 30% and less than 50% of the predicted value) group.

### HRCT examination

All the included patients underwent chest HRCT scan upon hospitalization. Siemens dual-source CT (SOMATOM Definition Flash) and Siemens 128-slice CT (SOMATOM Definition AS) were used for detection. And the CT scan parameters were set as following: average tube voltage 120 kV, the diameter of the tube was 120 mm, the reconstruction layer thickness was 1 mm. all the examination were performed in a supine position during scanning, and patients were told to held their breath after inhalation.

### The measurement of CSA

Three levels of images were selected and analyzed as following [16]: 1 cm at the upper edge of the aortic arch (upper lung field); 1 cm below the carina (middle lung field); and 1 cm below the right lower pulmonary vein (lower lung field). We used Image J 1.52 software for data analysis, and the steps were as following [17, 18]: (1) Segmenting the lung field with a threshold of 500 HU to 1 024 HU; (2) Converting into a binary image with a window of 720 HU; (3) The cross-sectional area of each blood vessel was defined as < 5 mm^2^, the midpoint range of the "analyze particle" function is set to 0.9 to 1.0; (4) The total cross-sectional area of the pulmonary small blood vessels with a total of 3 levels < 5 mm^2^ were calculated as CSA. In order to ensure the consistency of the data measurement, under the guidance of a chief physician with more than 20-year experience, the measurement was independently completed by two physicians, and the average value was taken as the final result.

### PA/A calculation

The PA/A calculation was conducted as following [19]: The diameter of the main pulmonary artery was measured at the bifurcation of the left and right pulmonary arteries, the maximum diameter of the ascending aorta was determined in the same CT plane, and the ratio of PA to A was calculated. If the diameter of the main pulmonary artery was inconsistent during the measurement, the larger diameter was taken as the diameter of the main pulmonary artery.

### Data collections

The general patient information and related clinical data of each included patient were collected and analyzed, including patients’ identity, age, concurrent complications, smoking history (smoking was defined as at least one cigarette a day), vital signs and arterial blood gas analysis upon admission, and the CSA and PA/A. Two authors independently collected above information.

### Statistical analysis

This present study used SPSS 21.0 statistical software for statistical processing. The normally distributed continuous data were presented as mean ± standard deviation, and the binary data was expressed as the number of cases. We had performed test for normality, and the difference between groups was compared by single factor analysis of variance, and the Tukey's HSD (honestly significant difference) post hoc test is used for pairwise comparison. And Spearman’s test was used for potential relation analysis. Besides, the curve on the receiver operating characteristics (ROC) were made on the related HRCT results, and the area under curve (AUC) was calculated for the diagnosis value. We calculated the cutoff value according to the maximum Youden index and find the best cut point. *p* < 0.05 was considered as statistically different in this present study.

## Results

### The characteristics of included patients

A total of 128 COPD patients were included finally, with 41 patients in mild group, 45 in moderate group, and 42 patients in severe group. As Table 1 presented. There were significant differences in the duration of COPD, smoking history, the PaO_2_, PaCO_2_, pH, and FEV1, FVC and FEV1/FVC among COPD patients with different severity (all *p* < 0.05). No statistical differences were found on the gender, age and number of current smokers among three groups (all *p* > 0.05).

**Table 1:** The characteristics of COPD patients with different severity

Groups	Cases	Male/female	Age (years)	Duration of COPD(years)	Smoking history (packyears)	Number of current smoker	PaO_2_ (mmHg)	PaCO_2_(mmHg)	FEV1(L)	FVC (%)	FEV1/FVC (%)	ph
Mild group	41	36/5	69.3 ± 3.94	3.5 ± 1.24	38.4 ± 5.18	35	73.8 ± 7.42	48.4 ± 3.17	1.23 ± 0.19	88.66 ± 8.03	51.03 ± 7.47	7.4 ± 0.38
Moderate group	45	38/7	68.9 ± 4.02	5.7 ± 1.93	45.2 ± 7.36	38	56.2 ± 6.46	60.2 ± 4.05	0.91 ± 0.18	79.58 ± 7.12	38.12 ± 9.12	7.3 ± 0.51
Severe group	42	34/8	69.4 ± 3.97	7.1 ± 2.16	37.7 ± 6.05	35	49.4 ± 5.03	76.5 ± 5.22	0.59 ± 0.12	63.94 ± 6.44	29.14 ± 8.05	7.2 ± 0.59
*p*		0.120	0.097	0.008	0.034	0.097	0.029	0.009	0.005	0.002	0.010	0.047

COPD, chronic obstructive pulmonary disease

### The CSA and PA/A

As Table 2 presented, there were significant difference in the CSA and PA/A among COPD patients with different severity (all *p* < 0.05). As the COPD condition worsens, CSA gradually decreases, while PA/A increases.

**Table 2:** The CSA and PA/A of COPD patients with different severity

Groups	Cases	CSA (%)	PA/A
Mild group	41	0.68 ± 0.12	0.83 ± 0.29
Moderate group	45	0.62 ± 0.15	0.88 ± 0.25
Severe group	42	0.55 ± 0.13	0.94 ± 0.30
*p*		0.013	0.009

CSA, cross-sectional area; PA/A, the ratio of pulmonary artery and aorta

### The potential related variables and COPD severity

As Table 3 indicated, the duration of COPD, smoking, PaO_2_, PaCO_2_ (r = 0.440), CSA, PA/A, FEV1, FVC and FEV1/FVC were correlated with the COPD severity (all *p* < 0.05).

**Table 3:** The Spearman’s analysis on the potential related variables and COPD severity

Variables	ρ	*p*
Duration of COPD	0.663	0.048
Smoking	0.507	0.020
PaO_2_	0.612	0.045
PaCO_2_	0.528	0.039
CSA	0.611	0.016
PA/A	0.494	0.011
FEV1(L)	0.516	0.010
FVC (%)	0.623	0.012
FEV1/FVC (%)	0.587	0.009

COPD, chronic obstructive pulmonary disease; CSA, cross-sectional area; PA/A, the ratio of pulmonary artery and aorta

### The correlation between CSA, PA/A and post BD FEV1

As presented in Table 4, both CSA, PA/A were correlated with post BD FEV1 (all *p* < 0.05).

**Table 4:** The correlation between CSA, PA/A and post BD FEV1

	ρ	*p*
CSA	0.614	0.037
PA/A	0.557	0.041

CSA, cross-sectional area. PA/A, the ratio of pulmonary artery and aorta

### CSA and PA/A for the diagnosis of severe COPD

**Fig. 1:**
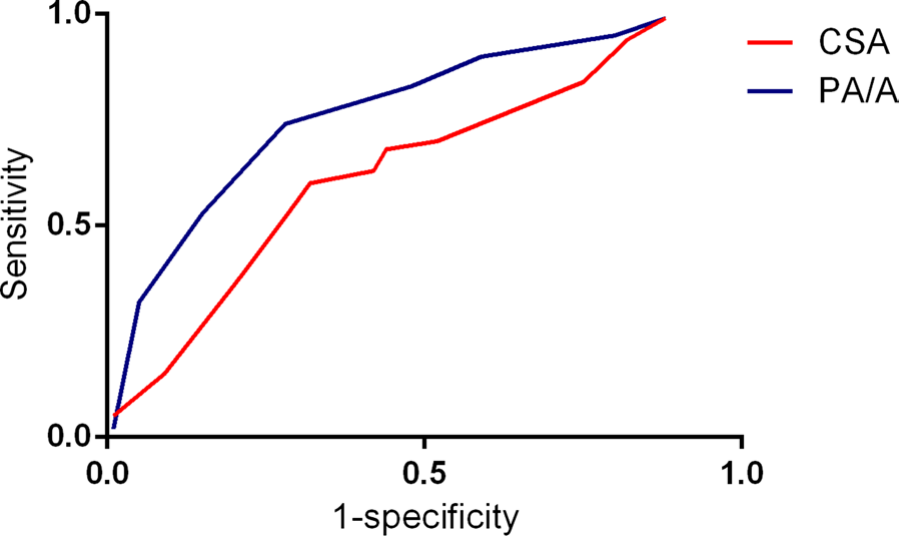
ROC of CSA and PA/A for the severity diagnosis of COPD

Figure 1 presented the ROC of CSA and PA/A for the diagnosis of severe COPD. As Table 5 presented, the cutoff value of CSA and PA/A for the diagnosis of severe COPD was 0.61 and 0.87 respectively, and the AUC of CSA and PA/A for the diagnosis of severe COPD was 0.724 and 0.782 respectively.

**Table 5:** The predictive value of CSA and PA/A for the severity diagnosis of COPD

Variables	Cutoff value	AUC	95% CI	Sensitivity (%)	Specificity (%)
CSA	0.61	0.724	0.637–0.799	79.25	81.03
PA/A	0.87	0.782	0.725–0.814	80.20	85.20

COPD, chronic obstructive pulmonary disease; CSA, cross-sectional area; PA/A, the ratio of pulmonary artery and aorta; AUC, area under curve

## Discussion

At present, COPD is one of the major public health problems in the world, and it is expected to be the fourth leading cause of death in the world by 2030 [20, 21]. It’s been estimated that more than 390 million people may have COPD in 2030 [22]. The pathophysiological changes of COPD include the involvement of lung parenchyma and pulmonary blood vessels [23]. On the one hand, the lung parenchyma is gradually destroyed, resulting in the loss of attachment of alveoli and small airways and the reduction of lung elastic retraction, which ultimately leads to emphysema [24]. On the other hand, long-term hypoxia, chronic inflammation and other factors lead to pulmonary vasoconstriction, pulmonary vascular remodeling, which shows that the proportion of pulmonary small blood vessels in the total area of the lung field also gradually decreases, resulting in pulmonary artery widening and pulmonary hypertension [25, 26]. Thus, the early detection on the changes of COPD is essential to the prognosis of patients.

Advances in chest HRCT scanning technology have enabled the accurate measurement and evaluation of changes in pulmonary blood vessels [27]. Pulmonary small blood vessels are usually defined as a single branch with a cross-sectional area of < 5 mm^2^. however, CT can also be biased if the patients do not follow instructions [28]. It has been reported that the CSA of COPD patients may be associated with the symptom, average pulmonary artery pressure and emphysema severity in COPD patients [29, 30]. The results of this present study have further confirmed that the CSA and PA/A are closely associated with the severity of COPD, and the COPD patients with CSA ≤ 0.61 and PA/A ≥ 0.87 have higher risks for severe COPD. For such patients, early warning and management measures should be taken.

Pulmonary vascular changes are one of the important mechanisms involved in the exacerbation of patients with acute exacerbations [31]. Clinically, patients with COPD can be divided into mild, moderate and severe three levels according to their clinical manifestations, arterial blood gas analysis and other indicators to guide clinical management strategies. The pathological changes of pulmonary blood vessels in patients with COPD mainly include the spasm and compression of pulmonary small blood vessels and the remodeling of blood vessel walls [27, 32]. And pulmonary artery widening is common and pulmonary hypertension may appear. Some scholars have observed that PA/A will also increase, and pulmonary artery widening is related to heart injury markers [33]. And the acute increase in pulmonary artery width returns to baseline after an acute exacerbation event. Due to long-term chronic hypoxia and chronic inflammation, the pulmonary blood vessels of COPD patients have undergone obvious pathophysiological changes, which are manifested by a significant reduction in the cross-sectional area of the pulmonary small blood vessels, especially as the disease worsens [34, 35]. Previous studies [36–38] have shown that during the year of follow-up, CSA subjects receiving regular and reasonable management have significantly increased CSA, especially among smoke quitters. This suggests that in patients with COPD, if they can be early detected on their pulmonary small blood vessel and, and with active and effective management, smoking ban, correction of hypoxia, acidosis, inflammation and other factors may restore the small blood vessels to their original shape to a certain extent [39]. Therefore, it is possible to avoid the occurrence of irreversible pathological changes of the pulmonary vessels, thereby improving the long-term prognosis of COPD patients.

Compared with lung function tests, HRCT does have some advantages. It can directly and objectively measure lung tissue through chest imaging data, and the accuracy is relatively higher. And the lung volume measured by chest CT computer software algorithm processing does not include the invalid content of gas in the nasopharynx, oral cavity, trachea, and airway [40]. Strictly speaking, lung function examination cannot avoid and exclude this part of gas (estimated about 100 ml), so the CT measurement is closer to the actual conditions. Furthermore, whether it is overall or local, unilateral or bilateral, chest CT can observe and accurately measure the lung volume, which is irreplaceable for lung function tests that can only measure the sum of bilateral lung volumes, especially it is a preoperative evaluation of patients who are about to undergo surgery [41, 42]. Furthermore, chest CT is simple and safe to perform for the deficiency of COPD patients with severe illness and patients with acute attacks who cannot cooperate with the lung function tests [43]. With the advent of the third-generation dual-source CT, the scan time of the chest HRCT is less than 3 s [44]. Even patients with poor lung function and unable to cooperate with forced exhalation and inhalation movements can successfully pass a few seconds of breath holding movements to complete the chest CT examination [45]. However, compared with lung function tests, HRCT still has some limitations. Pulmonary function examination can reflect the expiratory index such as the peak expiratory flow (PEF) by directly measuring the expiratory air flow, but HRCT cannot achieve it [46]. Thus, the combined use of HRCT and lung function test may be a better option for patients with COPD.

Several limitations must be considered in this present study. Firstly, the patients with severe COPD showed the worst blood gas constitution and the worst measures in imaging parameters such as CSA and PA/A even though they reduced their smoking. In this context, the patients are needed to be diagnosed at the earlier stage of the disease to stop the disease progression. Thus, further ROC analysis may help to diagnose even the mild COPD patients. Limited by sample size, it’s difficult for us to preform this analysis, future studies with larger sample size are warranted. Secondly, the larger diameter was taken as the diameter of the main pulmonary artery, there may be some data collection bias working towards a positive finding, the average values may be more appropriate to reduce the bias. Thirdly, smoking history is stated to vary across severity groups although it is not specified that the moderate group had the highest smoking intensity and the severe group the lowest smoking intensity, the raw data on the smoking history was very subjective, future studies with larger sample size are needed in the future. Finally, all COPD patients routinely underwent CT scan, it can be a bias in enrolled patients because other COPD patients who did not undergo CT were not enrolled.

## Conclusions

In conclusion, the decrease of CSA and increase of PA/A may be effective indicators for assessing the clinical severity of COPD patients, and COPD patients with CSA ≤ 0.61 and PA/A ≥ 0.87 have higher risks for severe COPD. Clinically, early management measures should be taken for those patients. However, the mechanism and management strategy for changes in pulmonary vascular parameters in patients with COPD are still unclear, and further experimental and clinical studies are needed.

## Data Availability

All data generated or analyzed during this study are included in this published article.

## Abbreviations

COPD: Chronic obstructive pulmonary disease
CSA: Cross-sectional area
PA/A: The ratio of pulmonary artery and aorta
HRCT: High-resolution computed tomography
ROC: Receiver operating characteristics
AUC: Area under curve
